# Biological properties versus solubility of endodontic sealers and cements

**DOI:** 10.2340/biid.v11.40863

**Published:** 2024-06-04

**Authors:** Inge Fristad, Sivakami Haug, Asgeir Bårdsen

**Affiliations:** Department of Clinical Dentistry, University of Bergen, Bergen, Norway

**Keywords:** Endodontics, biomaterials, biocompatibility, toxicity

## Abstract

Endodontic sealers and cements used in root canal treatment have different compositions and properties. Common to all materials is that their primary goal is to fill gaps and voids, making a permanent seal of the root canal system. Furthermore, aspects such as antibacterial properties, cytotoxicity, setting time, solubility and biocompatibility are also crucial and ought to be considered. Over the years, a shift in the view on the importance of these aspects has ocurred. Whereas the antibacterial properties were considered important when the technical factors in endodontics were less developed, the sealing ability and biocompatibility have later been considered the most critical factors. The introduction of tricalcium silicate cements and sealers has led to a renewed interest in material properties, as these cements seem to have good sealing ability and at the same time combine favourable antimicrobial effects with excellent biocompatibility. This review discusses how the various properties of root canal sealers and cements may conflict with the primary aim of providing a permanent seal of the root canal system.

## Introduction

A causative factor in endodontic disease is infection of the root canal system [1]. Eradication of biofilm and planktonic bacteria is therefore crucial for a successful endodontic treatment outcome [2]. The primary infection control is achieved during chemo-mechanical cleaning and shaping of the root canal, whereas the endodontic materials should provide and secure a long-term seal to prevent secondary infection of the root canal system.

It has been extensively documented that the quality of the root canal filling is closely related to a successful endodontic outcome, and that the choice of biomaterial may be of less importance [3–5]. Furthermore, the prognosis of endodontic treatment of teeth with vital pulp is generally higher than what is found for teeth with preoperative apical periodontitis (3, 5). This may depend on persistent intracanal infection or by factors such as extra-radicular infection, foreign-body reactions, or cysts [6]. It has also been documented that late failures in most cases are related to non-endodontic factors.

**Figure 1:**
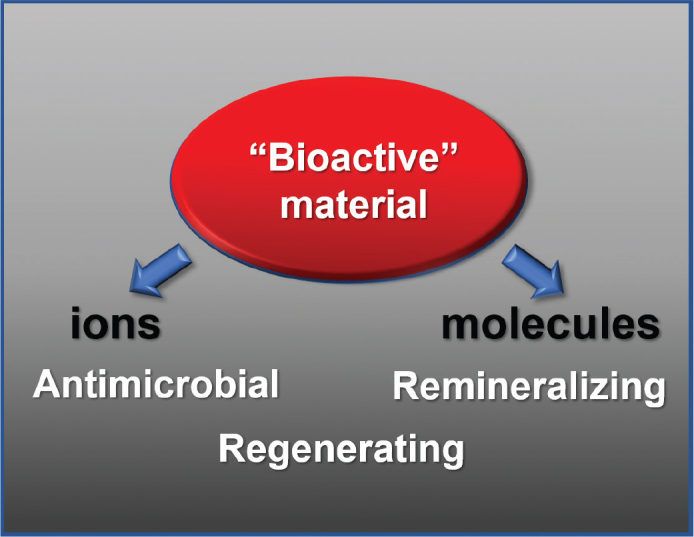
Antimicrobial, remineralising and regenerating properties of a bioactive material often rely on solubility of the material. A root filling material should ideally not dissolve, implying an inert material.

In clinical use of endodontics biomaterials, some conflicting issues should be kept in mind. Firstly, the material should be stable over time to provide a long-term seal of the root canal system. This may conflict with solubility of the material needed for an intended release of bioactive molecules, either in the form of a long-term antibacterial effect or as a long-term bio-stimulating or bioactive material that is beneficial for the host tissue (Figure 1). Secondly, biomaterials used in endodontics are intended to set in a moist environment. Most of the commonly used endodontic sealers and cements are cytotoxic during setting, whereas cytotoxicity is generally reduced over time [7]. Therefore, an initial cytotoxic effect that can be beneficial for the sealer’s ability to have an antibacterial property at an early stage may be lost over time. The term bioactive materials are widely used in various settings, including effects on mineralisation, pH buffering properties, or with antimicrobial, antifouling, or biofilm modulating abilities. It has been questioned whether these effects rely on bioactivity or whether the effects are mediated by simple chemistry or toxicity [8]. Tissue engineering strategies, on the other hand, aim to regenerate destroyed or lost tissue. In these applications, the biomaterials should ideally act as a scaffold that is degraded and gradually replaced by host tissue. When the tissue is finally regenerated and healed, the biomaterials have completed their mission. Endodontic materials used for vital pulp treatment have some of these characteristics. For example, when a successful healing has occurred after pulp capping, with completion of a dentine bridge, the material has achieved some of its primary functions.

Biological interactions can be affected by cytotoxicity, cytocompatibility, cell plasticity, differentiation potential, and bioactive properties [9]. Although cytotoxicity and biocompatibility are interrelated, cytotoxicity is defined as toxic effects of a material on vital tissue [10], whereas biocompatibility is compatible and harmless properties on vital tissue [11].

Antibiotics are different from other antibacterial compounds, as they selectively eliminate bacteria, leaving the host cells unaffected. However, the risk of bacterial resistance makes them controversial in clinical use other than in acute infection control [12]. In tissue engineering strategies, however, antibiotics are often used to control infection during the initial healing stage.

**Figure 2:**
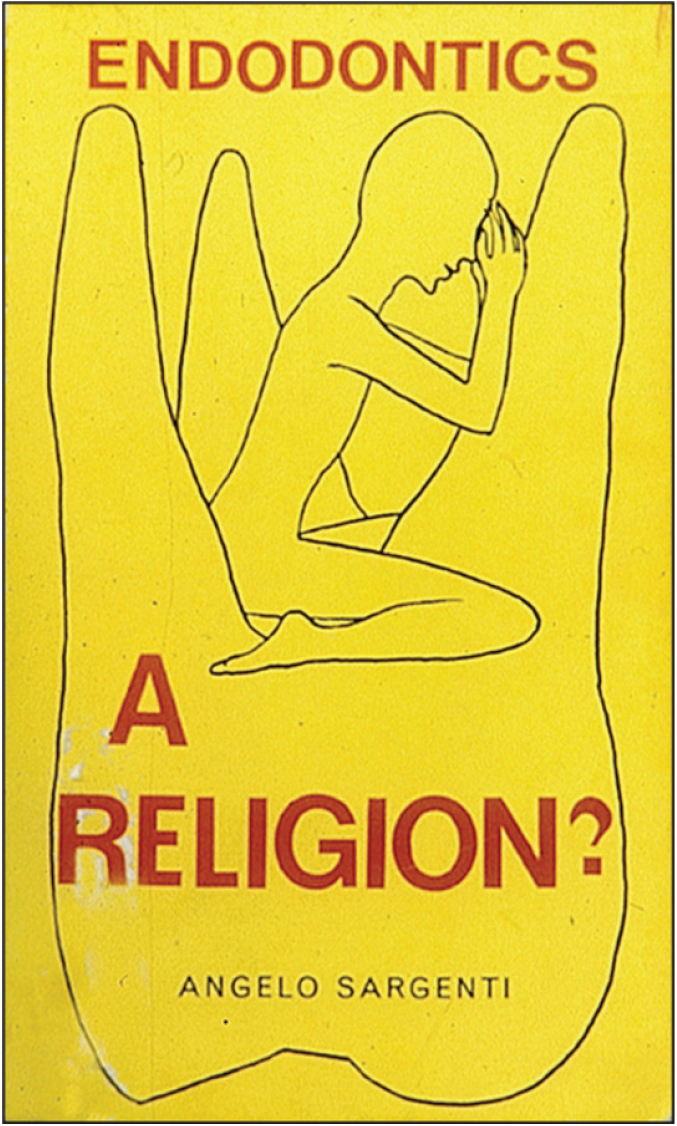
The practical antiseptic approach, illustrated by a citation from a pocketbook by Angelo Sargenti: ‘A single reamer of appropriate size can be used. The canal is filled with N2 Permanent pasta, and insertion of gutta-percha is not needed. Since bacteria are not able to live in contact with N2, irrigation with antiseptic solutions and bacterial control is not needed. Isolation with cotton rolls is sufficient’.

Endodontics has gradually developed into a discipline based on biological principles, being cautious with use of materials that may have adverse effects. However, over the years some have argued towards using a practical approach with simple cleaning methods and strong antiseptics to overcome infection of the root canal system (Figure 2). In summary, one may argue that empirical formulae with antibacterial additives were clinically relevant and successful when aseptic principles and canal debridement techniques were less well developed and advanced. On the other hand, current aseptic methods, often followed by interappointment disinfection and relative inert filling materials, are superior to methods relying solely on antibacterial components of the material [13]. However, the rationale and clinical usefulness of antibacterial additives have been and are still controversial [14, 15].

At present, endodontics relies on the biological principles of chemo-mechanical preparation to control the infection before a permanent endodontic material is placed to secure a long-term seal of the root canal system. At the same time vital pulp treatment, intended to preserve the tooth with intact vital pulp tissue, is encouraged. The properties of the biomaterials are in these applications critical, as they ideally should have both bacteriostatic properties, at least during the healing phase, and at the same time being able to stimulate hard tissue formation to preserve the vital tissue.Considering the aforementioned points, it may be beneficial that biomaterials used in endodontics have antibacterial properties, at least during the initial setting. The endodontic material should also be stable to secure a long-term seal of the root canal system. At the same time, bioactive properties that promote healing may be critical in some clinical applications. The present aim is to give an overview of biological properties, with focus on antibacterial aspects of common sealers and cements used in endodontic treatment, and at the same time keeping in mind the long-term stability which is important when endodontic biomaterials are used in the clinic.

## Cytotoxicity, biocompatibility, and bioactivity versus stability

**Figure 3:**
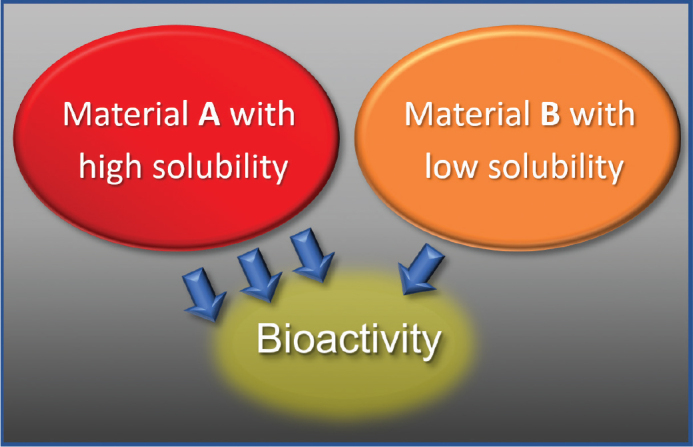
Bioactive properties rely on initial setting and/or solubility of the material over time. A) Bioactive material. B) Inert material.

**Figure 4:**
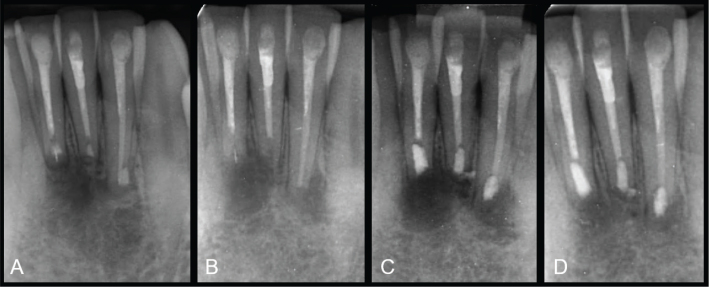
Retrograde fillings with IRM in lower front teeth in 2009 (A), follow-up in 2020 with loss of contrast of the retro-fillings and secondary infection (B), postoperative radiograph with TotalFill BC Putty (FKG, Switzerland) 2021 (C), and follow-up 5 months later with healing (D).

**Figure 5:**
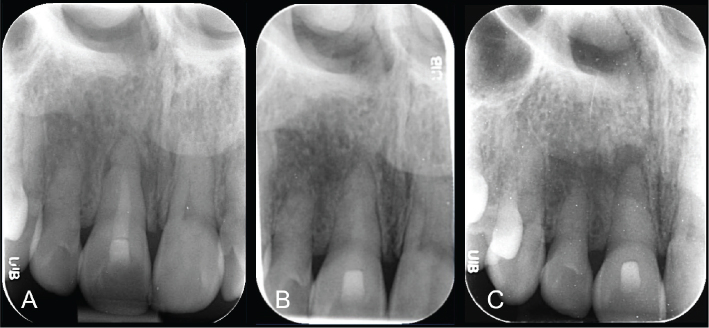
Trauma in tooth 11 with root fracture and radiolucency in the fracture line. After cleaning and shaping, the root canal was filled with Biodentine (Septodont, France) in the coronal fragment. Final root filling in 2013 (A), follow-up with healing in 2015 (B), and with infection in the apical fragment 10 years postoperatively (C). Note the loss of radiopacity of the material, with disintegration of the material confirmed during apical surgery.

Root canal sealers and cements commonly used in endodontic practice are presented in Table 1. The antibacterial properties of the various endodontic materials are closely related to their cytotoxicity, often considered as ‘adverse effects’ of biomaterials [16]. The cytotoxic effects of endodontic materials are often of short-term duration, connected to the setting reaction of the materials [13]. The widely used epoxy sealer AH26 (Dentsply Sirona, Germany), later replaced by AH Plus (Denstply Sirona, Germany) with reduced amounts of formaldehyde release during setting, is relatively cytotoxic and antimicrobial during setting, but after setting one of the most inert sealers [13]. At the same time AH Plus is shown to be stable with low solubility. Salicylates utilises the bioactive and antibacterial properties connected to calcium-hydroxide, a property relying on release of hydroxyl ions, resulting in an alkaline environment [17]. The salicylate sealer Sealapex (Kerr, USA) has shown a prolonged bio-stimulating and antibacterial effect due to release of hydroxyl ions, but at the expense of long-term stability because of solubility of the material [17, 18]. Hence, the bio-stimulating and antibacterial properties of various sealers seems to be closely related to the solubility of the sealers [17]. Apexit (Ivoclar Vivadent, Lichtenstein) is another example in the salicylate group, that is less soluble than Sealapex, but at the expense of bioactivity [19]. The new bioceramic sealers also have bio-stimulating and antibacterial effects based on the alkaline environment. The combination of the alkaline bio-stimulating environment and the ability to set in a wet environment, makes these materials ideal for applications in pulpotomy, apexification and perforation repair procedures. To illustrate the connection between cytotoxicity, biocompatibility, bioactivity and stability, a study showed that AH Plus had the lowest solubility at 2 and 7 days whereas the salicylate sealers Sealapex and MTA Fillapex (Angelus, Brazil), and the experimental bioceramic sealer MTA-S (Araraquara, Brazil) had significantly higher solubility in increasing order [17]. The pH values for salicylates and the bioceramic sealer were around 10, compared to 7 for AH Plus after 2 days. Figure 3 illustrates that the bioactive properties may affect the stability of the material, whereas Figures 4 and 5 show disintegration of root filling materials seen after long term follow-ups. Figure 4 is an illustration of the intermediate cement IRM (Dentsply DeTray, Germany) that has been used as a retro-filling material in surgical endodontics. According to the manufacturer, IRM is considered a temporary cement, intended to last for up to 1 year [20].

**Table 1:** Root canal sealers and cements based on setting reaction.

Acid-base reaction	Chelate formation		Polymer formation by addition reaction		Polymer formation by radical polymerisation	Hydration
Glass-ionomers	Salicylates	Zinc oxide- eugenol Zinc oxide- fatty acids	Silicone	Epoxy resin	Methacrylate resin	Bioceramics (hydraulic calcium silicate cements)

## Sealers and cements used in endodontic practice

### Sealers based on chelate formation

*Zink oxide eugenol (ZOE)-based sealers* were among the first sealers on the market. ZOE-based sealers are generally regarded as toxic or antibacterial, particularly in combination with antibacterial additives [21]. A recent review article found moderate evidence for strong antibacterial effects of freshly mixed ZOE-based sealers against *E. faecalis*, whereas most studies reported no antimicrobial activity for 2–7-day aged samples of eight tested sealers [22]. Only four studies reported positive effects of three sealers in this category. Taken together, there was conflicting evidence of a long-term antibacterial effect of ZOE-based sealers. However, most of the formaldehyde and paraformaldehyde-containing sealers belong to the ZOE-based sealers, and these sealers showed the strongest antibacterial properties in a study comparing sealers and paste filling materials [13, 23]. The same study also showed that the antibacterial effects were dramatically reduced for all sealers over time. Canals-N (Showa Yakuhin Kano, Japan) is an example of a non-eugenol sealer, where fatty acids replace eugenol as a chelating agent. Canals-N was less toxic than the eugenol-containing counterpart Canals (Showa Yakuhin Kano, Japan) [24].

*Salicylates or calcium hydroxide-containing sealers* were introduced early in the 1980s based on the favourable effects of calcium hydroxide on hard tissue formation in teeth. Pure calcium hydroxide exerts its effect through leakage of calcium and hydroxyl ions to the surrounding tissues [25–27].

As the main role of root canal sealers is to be stable and to fill gaps, solubility, leakage, and adhesion are prime concerns. Unless calcium and hydroxyl ions are released from the sealer, it will not have the expected stimulating effects on surrounding tissues. This has raised questions regarding the antibacterial and therapeutic effects of calcium hydroxide-containing sealers. Although Sealapex has shown high success rates for pulpectomies [28, 29], an animal study showed disintegration of the sealer with ingrowth of connective tissue, with sealer particles located in cells at some distance from the sealer sample [30]. Furthermore, Apexit sealer showed high solubility compared to the epoxy resin sealer AH Plus and the ZOE-based sealer Tubli-Seal (Kerr, USA) [31]. The antibacterial effects of salicylate sealers have been questioned. Although Sealapex and Apexit have shown increased pH in aqueous solutions [32], dentinal tubule penetration tests have shown that these sealers are less effective in killing bacteria than resin and eugenol-based sealers [33, 34]. MTA-Fillapex is basically a salicylate resin-based sealer that contains 15% MTA powder and should not be regarded as a tricalcium silicate sealer [35]. The solubility of MTA-Fillapex is therefore also a concern [17].

### Sealers based on acid-base reaction

*Glass ionomer cement (GIC)* is an acid base reaction between a basic fluoro-alumino-silicate glass powder and polycarboxylic acid in the presence of water. As a sealer used in endodontics, modifications have been performed to extend the working time and increase the radiopacity [36, 37]. These modifications resulted in the first commercially available sealer Ketac-Endo (3M ESPE, USA) in 1991. Antibacterial properties of the Glass ionomer-based cements are believed to rely on a combination of fluoride release [38], acidity [39] and zinc components [40]. GICs are relatively inert, as they caused a mild inflammatory reaction that diminished progressively, when compared to ZOE-based sealers [41, 42]. The biocompatibility is further supported by the inhibitory effect of Ketac-Endo on osteoclastic activity [43]. Although the antibacterial properties of GICs are limited, additives like resin, as used in Vitrebond, make them more antibacterial due to toxic agents released during setting [44]. Also, silver additives and modifications such as surface pre-reacted glass-ionomer fillers may increase their antibacterial properties [45]. GICs are sensitive to moisture during the endodontic procedure, which is not always easy to achieve in the apical region. In addition, long term studies on GICs are limited. Despite this, a study from 1995 found that the healing rate was in line with studies performed with other sealers [46]. A finding was also that surplus filling material was not resorbed in the periradicular area, indicating low solubility.

### Resin-based sealers

Among resin-based sealers, the best-known products are Diaket (ESPE, Germany), AH26 and its successor AH Plus. Advantages of resin-based sealers include antibacterial action, adhesion, long working time, ease of mixing, and very good sealing ability. Its disadvantages are staining, toxicity when unset, and some solubility to oral fluids.

Diaket, a is a resin-reinforced chelate formed between zinc oxide and a small amount of plastic dissolved in the liquid B-diketone. It is a very tacky material, it contracts slightly while setting, which is subsequently negated by uptake of water. Its sealing efficacy is good. The Diaket polyvinyl resin sealer shows greater strength and improved resistance to disintegration in water than ZOE-based materials. No adhesion to root dentin could be shown by this material, and flow is comparatively low [47, 48].

AH26 is an epoxy resin glue, and its base is biphenol A-epoxy. The catalyst is hexamethylene-tetramine (methenamine). It also contains 50% bismuth oxide for radiographic contrast. As AH26 sets, traces of formaldehyde are temporarily released, which initially makes it antibacterial. AH26 has been known for its good physiochemical properties such as dimensional stability, low contraction, sealing ability and flow [47, 49, 50]. High values for flow, strength, radiopacity, and adhesion to dentin were shown with AH26. This was the only commercial root canal sealer that gave definite adhesion to root dentin maintained in the presence of water. AH26 is not sensitive to moisture and will even set under water. The methenamine gives off formaldehyde as it sets, and this has been one of its major drawbacks.

AH Plus and ThermaSeal Plus (Dentsply Sirona, Germany) were formulated with a mixture of amine that would allow for polymerisation without the unwanted formation of formaldehyde, but with all the advantages of AH26, such as increased radiopacity, low solubility, slight shrinkage, and tissue compatibility. AH Plus is an epoxy-bisphenol resin that also contains adamantine. AH Plus comes as a two-paste system, unlike the liquid-powder system of AH26. AH Plus has a working time of 4 h and a setting time of 8 h.

AH26 has been shown to have good antibacterial properties [51]. Freshly mixed AH Plus exhibits high antibacterial properties, which reduces at 1- and 3-day intervals [52, 53]. Diaket sealers have mild antibacterial activity against *E. faecalis* compared with AH26 sealers. The results after 48 and 72 h showed that the antibacterial effects of the root canal sealers decreased slightly with time [54]. A systematic review reported that there is moderate evidence in favour of strong antibacterial activity of freshly mixed epoxy resin-based sealers [22].

Formaldehyde-containing sealers, such as AH26 are highly toxic [55]. The highest amount of formaldehyde release is in the freshly mixed sealer, and the amount of formaldehyde released goes down after 48 h, and after 2 weeks the amount released is insignificant [56]. In an *in vitro* study, measuring cytotoxicity of sealers, Diaket showed a moderate cytotoxic effect whereas AH26, surprisingly, showed comparably low cytotoxicity [57]. AH Plus has been shown to be less cytotoxic than AH26, but both cause a dose-dependent increase in genotoxicity [21].

### Silicon-based sealers

Silicone-based sealers utilise the same qualities as caulking compounds used in household constructions in kitchen and bathroom structures, providing adhesion, a moisture-resistant seal, and stability [58].

Lee Endo-Fill (Lee Pharmaceutical, CA) has a rubbery consistency when set. Initially, the manufacturer recommended that it should be injected into the canal as the sole sealer. The base of Lee Endo-Fill is heavily loaded with bismuth subnitrate as a radio-pacifier. The active ingredients are hydroxyl-terminated dimethyl polysiloxane, undecylenic acid, benzyl alcohol and hydrophobic amorphous silica. The catalysts are ethyl orthosilicate, polydimethylsiloxane, and catalyst intermediate. Advantages of Lee Endo-Fill are its ease of preparation (it is a paste-liquid to be spatulated) and adjustable working time, low working viscosity, and rubbery consistency. It is as easy to remove as gutta-percha. Its disadvantages are sensitivity to humidity and the requirement of a dry canal. It also shrinks slightly upon setting but has the ability to flow into open dentine tubules.

RoekoSeal (Coltene/Whaledent, USA) is a polydimethylsiloxane-based material. RoekoSeal is reported to polymerise without shrinkage and utilises platinum as a catalyst [56]. It was noted that root fillings with RoekoSeal were leaking more than others, but after 21 days, the situation was reversed. The slow setting properties of this material could be an explanation for diminished leakage. However, clinical follow-up studies show that RoekoSeal performs just as well as Grossman’s ZOE sealer (Ultimate Dental, USA) [59].

GuttaFlow (Coltene/Whaledent, USA) is a polyvinylsiloxane with finely milled gutta-percha particles added to the RoekoSeal sealer. GuttaFlow also contains silicone oil, paraffin oil, platin catalyst, zirconium dioxide, nano-silver as a preservative, and a colouring agent. It is also eugenol free. It is a cold flowable gutta-percha filling system for the obturation of root canals. GuttaFlow is triturated in its cannula and passively injected into the canal and then used with single or multiple gutta-percha points. GuttaFlow2, an evolution of its predecessor GuttaFlow, is also a cold flowable system combining gutta-percha powder with a particle size of less than 30 μm and sealer. Both GuttaFlow and GuttaFlow2 are silicone-based endodontic sealers that differ in the form of the silver particles used. GuttaFlow Bioseal (Coltene/Whaledent, USA) is a formulation of polydimethylsiloxane with gutta-percha powder combined with calcium silicate particles.

Silicon-based sealers are virtually non-toxic and the least irritating sealer on the market. GuttaFlow exhibits lower cytotoxicity than AH Plus [60]. GuttaFlow exhibits no antibacterial properties [52]. Interestingly, the antibacterial activity of GuttaFlow Bioseal increased as the material aged up to 4 weeks, demonstrating initial antimicrobial capacity [61].

### Urethane Methacrylate Sealer

EndoREZ (Ultradent, USA) is a hydrophilic UDMA resin-based sealer with an easy delivery system, good canal wetting properties and flow into dentinal tubules. EndoREZ is introduced into the canal with a narrow 30-gauge Navitip needle. A single gutta-percha point technique or the lateral compaction technique may be used for obturation. In scanning electron microscopy (SEM) examination, a high mean maximum adaptation of methacrylate resin-based sealer EndoREZ has been shown [50, 62, 63].

A study of the response of EndoREZ on bone, revealed that at 10 days after placement, the amount of reactionary bone formation in direct contact with EndoREZ was significantly reduced, and the number of inflammatory cells next to the EndoREZ sealer was relatively high. However, after 60 days, no differences were noted between the experimental and control groups. This indicated that the sealer produces an initial toxic response like that of many other sealers [64].

The penetration of these sealers into dentinal tubules appears to depend on the chemical and physical properties of the material. The epoxy resin-based sealer AH26 and the methacrylate resin-based sealer EndoREZ displayed deeper and more consistent penetration compared with the ZOE-based sealer [50].

A systematic review on the antibacterial activity of methacrylate sealers reported mild to no inhibition of *E. faecalis* [22].

### Bioceramic-based sealers

Mineral trioxide aggregate (MTA), a Portland cement-based material, was launched as a new endodontic material in the early 1990s. MTA is the most studied material in this group, and is often used as a reference material when studying properties of the bioceramic materials [65]. This material was introduced as a root-repair material (retrograde material, orthograde apical and lateral barriers and pulp preserving procedures) and showed good biocompatibility and sealing abilities [66, 67]. New bioceramic materials have later become available as root-repair materials, although with slight differences in handling properties, surface characteristics, solubility, and calcium release. Based on their favourable biocompatible properties, bioceramic cements were also utilised in the development of endodontic sealers. However, to be used as sealers in root canal treatment, the fluidity needed improvements.

Bioceramic sealers are based on silicate and therefore classified as hydraulic calcium silicate cements [68]. As sealers, they must comply to ISO specification 6876:2012 [69]. Bioceramic sealers have gained popularity due to their assumed advantageous properties, as seen for the root-repair materials, including biocompatibility, sealing ability, dimension stability, moisture tolerance and antibacterial properties.

A former distinction in the use of bioceramic endodontic materials was related to the clinical context in which they were used, either used intra-coronal for pulp protection and barrier for regenerative procedures, intra-radicular for sealing of the root canal space, or extra-radicular as root end fillers and repair of perforations. Although the applications are different, many of the biological properties are closely related. A classification of hydraulic calcium silicate cements based on their contents as proposed by Camilleri in 2020 [68], are given in Table 2. This classification depends on the setting reaction, either being a material that is activated when mixed with water or a one-component material in a non-aqueous vehicle that utilises humidity in the root canal.

**Table 2:** Classification of hydraulic calcium silicate cements based on their contents as proposed by Camilleri [68].

Type	Cement	Additives	Water	Commercial examples
1	Portland cement	No	Yes	ProRoot MTA
2	Portland cement	Yes	Yes	MTA Angelus, MTA HP
3	Portland cement	Yes	No	BioC (Angelus)
4	Tricalcium/dicalcium silicate	Yes	Yes	Biodentine, BioRoot
5	Tricalcium/dicalcium silicate	Yes	No	TotalFill

A selection of bioceramic sealers on the market, combined with studied properties, are presented in Table 3. Most recent hydraulic calcium silicate cements are in category 5, that is, tricalcium/dicalcium silicate sealers with additives and in a ready to use form (premixed) (Table 3). Table 4 gives an overview of several properties studied for different bioceramic sealers.

**Table 3:** Some available bioceramic sealers and their classification according to Camilleri [68].

Classification	Material	Manufacturer	Main component
***powder/liquid***			
4	Bio MM	St. Joseph University, Beirut, Lebanon	Tricalcium silicate
4	BioRoot RCS	Septodont Corp., France	Tricalcium silicate
2	Endo CPM sealer	EGEO, Argentina	Tricalcium silicate
2	Neo MTA Plus	NuSmile Avalon Biomed, USA	Tricalcium silicate
2	ProRoot ES	Dentsply Tulsa, USA	MTA-based/calcium silicate
	Tech BioSealer	Profident, Kielce, Poland	
***paste to paste***			
3	MTA Fillapex	Anguls, Brazil	MTA-based
4	Nishika canal sealer BG	Nippon Shika Yakuhin Co., LTD, Japan	Calcium silicate
***premixed***			
5	AH Plus BC sealer	Dentsply Sirona, Germany	Calcium silicate
3	Bio C sealer	Angelus, Brazil	Portland cement/tricalcium silicate
5	CeraSeal	Meta Biomed, Korea	Calcium silicate
5	EndoSeal MTA	Maruchi, USA	Pozzolan cement/ tricalcium silicate
	EndoSeal TCS	Maruchi, Wonju, Korea	Tricalcium silicate
5	EndoSequence BC sealer	Brassler, USA	Tricalcium silicate
5	iRoot SP	Innovative BioCeramix Inc., Canada	Calcium silicate
5	Nano-ceramic Sealer	B&L Biotech, Fairfax, VA, USA	Calcium silicate
5	Sealer Plus BC	MK Life Produtos Medical e Dental, Porto Alegre, Brazil	Calcium disilicate
5	TotalFill BC sealer	FKG Dentaire Sarl, Switzerland	Tricalcium silicate
5	Well-Root ST	Vericom, Korea	Tricalcium silicate

**Table 4:** Some bioceramic sealers for intra-canal use and relevant properties studied with references.

Classification*	Material	Properties studied	References
***powder/liquid***			
4	Bio MM	Physio-chemical properties	[70]
4	BioRoot RCS	Antimicrobial effect Biocompatibility/cytotoxicity Mineralisation activity Physio-chemical properties	[17, 70–74] [72, 75–78] [75, 79] [70, 80]
2	Endo CPM sealer	Biological properties Resistance to push-out Antimicrobial properties Sealing ability	[81] [81] [82] [83]
2	Neo MTA Plus	Biocompatibility/cytotoxicity Mineralisation activity	[84] [84]
2	ProRoot ES	Sealing ability Biocompatibility/cytotoxicity Mineralisation activity	[85] [76, 86, 87] [87]
	Tech BioSealer	Physio-chemical properties	[88]
***paste to paste***			
3	MTA Fillapex	Biocompatibility/cytotoxicity Resistance to push-out Antibacterial effect Physio-chemical properties Sealing ability	[76–78, 89–92] [81] [17, 73, 82] [17] [93]
4	Nishika canal sealer BG	Sealing ability Biocompatibility/cytotoxicity Mineralisation activity Physio-chemical properties	[94] [95] [95] [95]
***premixed***			
5	AH Plus BC sealer	Physiochemical properties Biological properties	[96] [96]
3	Bio C sealer	Biocompatibility/cytotoxicity Mineralisation activity Physio-chemical properties	[97] [97] [98, 99]
5	CeraSeal	Sealing ability Biocompatibility	[94] [100]
5	EndoSeal MTA	Sealing ability Biocompatibility/cytotoxicity Mineralisation activity Physio-chemical properties Biological properties	[94] [75, 101] [75] [95] [95]
	EndoSeal TCS	Biocompatibility/cytotoxicity	[100]
5	EndoSequence BC sealer	Biocompatibility/cytotoxicity Physico-chemical properties Sealing ability Antimicrobial activity Mineralisation activity	[72, 75, 87, 90, 102–104] [103] [105, 106] [104, 107–109] [75, 87]
5	iRoot SP	Biocompatibility Sealing ability Antimicrobial activity Physio-chemical properties	[110] [110, 111] [53, 112] [113]
5	Nano-ceramic Sealer	Biocompatibility/cytotoxicity	[101]
5	Sealer Plus BC	Biocompatibility/cytotoxicity Physio-chemical properties Antimicrobial activity	[92, 114] [98] [114]
5	TotalFill BC sealer	Antimicrobial efficacy Biocompatibility/cytotoxicity Mineralisation activity Physio-chemical properties	[71, 74, 80, 114, 115] [97, 114] [97] [98, 99]
5	Well-Root ST	Biocompatibility/cytotoxicity Physio-chemical properties Biological properties	[86, 101] [95] [95]

* Based on the classification by Camilleri 2020 [68].

As hydraulic calcium silicate cement, these sealers need water or other liquid to set. Water-based sealers like BioRoot RCS become fully set after mixing, but pre-mixed/ready to use sealers do not set in dry environment [116]. Based on this, remnants of irrigation solutions or humidity in the root canal, may influence the setting and quality of the seal. The use of sodium hypochlorite irrigation demonstrated a potentiated antimicrobial effect of these sealers [108]. However, calcium chelators such as ethylenediaminetetraacetic acid (EDTA) may negatively affect the mineral interaction with hydraulic sealers due to removal of minerals and exposure of collagen [117, 118], and chlorhexidine may directly deteriorate the physical characteristics of the sealer [119]. A study by Donnemeyer et al. showed that EDTA had a negative impact on bioceramic sealers and their push-out bond strength [120].

Biocompatibility is measured in various terms like cell viability, inflammatory response, osteogenic potential, cell attachment and morphology. A study by Oh et al., comparing CeraSeal, EndoSeal TCS and AH Plus, showed that the calcium silicate-based sealers had better cell viability than AH Plus, and AH Plus had a higher expression of inflammatory markers like IL-6 and IL-8. AH Plus had the lowest osteogenic potential, with EndoSeal TCS showing the best result based on expression of osteogenic markers. Cell proliferation was also enhanced for the calcium silicate-based sealers. In conclusion, calcium silicate-based sealers showed better biocompatibility and less cytotoxic effects compared to AH Plus [100]. Another study showed that pre-mixed calcium silicate-based sealers exhibited variable setting time and solubility with a decreasing inflammatory response [121]. The moist-dependent setting time with high solubility poses a concern for the clinical use of these pre-mixed sealers.

A study by Kahlil et al., comparing BioRoot RCS, Bio MM and AH Plus, showed that Bio Root RCS had the shortest setting time followed by Bio MM and AH Plus. Both Bio MM and Bio Root RCS had inferior flow than AH Plus, but with a higher film thickness. The radiopacity for AH Plus had a higher score than the bioceramic sealers. The pH value for AH Plus after one day was 8.4 and 8.7 after 28 days. Bio MM had a pH of 10.9 after 1 day, raising to 11.9 after 28 days. Similar results for Bio Root RCS were 12.1 and 12.7 after 1 day and 28 days, respectively [70].

A recent study comparing Bioceramic Sealer, EndoSequence BC Sealer and AH Plus showed that the bioceramic sealers presented adequate properties regarding physiochemical and biological properties. However, AH Plus Bioceramic Sealer had significantly higher release of calcium ions than AH Plus, but significantly lower than EndoSequence BC Sealer [96]. The release of calcium ions may be related to the solubility of the sealer. Another study comparing volumetric loss of AH Plus and EndoSequence BC Sealer showed that the volumetric substance loss after 30 days was close to twofold higher *in vivo* and fourfold higher *in vitro* for EndoSequence BC Sealer [122]. A recent study comparing AH Plus Bioceramic, Bio-C sealer and Bio-C Ion+ and AH Plus, showed that AH Plus Bioceramic was the most soluble sealer followed by Bio-C sealer, Bio-C Ion+ and AH Plus [123]. Donnemeyer et al. showed that AH Plus Bioceramic sealer and TotalFil BC-sealer were associated with significantly higher solubility than AH Plus over 1 month in distilled water and 4 months in phosphate-buffered saline (*p* < 0.05) [124]. This solubility is supported by the study by Raman & Camilleri [125], comparing three single syringe hydraulic cement-based sealers. They concluded that the tested bioceramic sealers had a higher solubility then given by the ISO standard for root canal sealing materials [69].

Adaptation to the canal wall and penetration depth of the sealers CeraSeal, EndoSeal MTA, Nishika Canal Sealer BG and AH Plus have been studied [94]. Three levels, apical (3 mm from apex), middle (6 mm from the apex) and coronal (9 mm from the apex) were measured. Results showed that the Nishika Canal Sealer BG had significantly less gaps in the apical third compared to EndoSeal MTA. At the middle third, EndoSeal MTA showed more gaps than both Nishika Canal Sealer and AH Plus. At the coronal third, there were no significant differences in sealer adaptation. For the penetration depth, there were significant differences only at the coronal level. Nishika Canal Sealer BG had higher penetration depth than AH Plus and EndoSeal MTA, whereas CeraSeal showed higher penetration depths than EndoSeal MTA [94].

Apart from providing a physical seal, the antibacterial properties of the bioceramic materials in the root canal system are dependent on solubility with release of calcium ions and high pH (alkalinity). Upon setting, bioceramic materials release calcium hydroxide, which leads to an elevated pH in the environment. Studies have shown that bioceramic sealers may have a positive effect on the eradication of *E. faecalis*. The study by de Souza et al. showed that AH Plus Bioceramic and EndoSequence BC Sealers eradicated *E. faecalis* after 24 h of direct contact [96]. Similarly, MTA Fillapex reduced the number of bacteria in biofilms [17].

Good dimensional stability for bioceramic sealers may simplify the obturation technique with less gutta-percha needed in the shaped canal(s) [126, 127]. A retrospective study by Chybowski et al. showed a success rate of 90.9% for BC Sealer and the single cone technique [128]. Further, two non-random prospective studies using the single-cone technique and BioRoot RCS, achieved a 1-year success rate of 90% – 97% [129, 130].

The antibacterial properties of endodontic sealers and cements are in general related to their sealing ability. Gutta-percha points coated with bioceramic nanoparticles may improve the sealing between the gutta-percha and the hydraulic calcium silicate-based sealers [113]. A uniform mono block within the root canal may also increase the fracture resistance of the root [113].

## Conclusion

The various requirements for endodontic sealers and cements should not be seen isolated and independently. In the short term, an antibacterial effect may help eliminate residual bacteria that are left after mechanical and chemical cleaning of the root canal. This effect is usually connected to the setting of the endodontic sealers and cements, and is gradually reduced and lost over time. In a long-term perspective, the stability of the material may be the most critical factor. Many of the traditional sealers and cements have proved to secure a stable and long-term result in extended follow-up studies [131, 132]. The new bioceramic materials have shown favourable biological properties and have shown antibacterial effects based on their alkalinity. However, many of the bioceramic sealers exceed the solubility range set by ISO 6876:2012, which may have an impact on the long-term outcome [125]. In addition, results have shown that the solubility among the bioceramic sealers are higher than many of the traditional sealers, and that the solubility varies between the different materials [123, 124]. Especially, the variable moist-dependent setting time with high solubility poses a concern for the clinical use of these pre-mixed bioceramic sealers [121]. Long term follow-up studies are therefore advocated for the new bioceramic sealers. Particularly, solubility is identified as a possible concern for the long-term seal of the root canal.

## Data Availability

Data sharing is not applicable to this article as no new data were created or analysed in this study.

## Key messages

Biological properties of endodontic materials should not be viewed isolated and should, as a minimum, secure a long-term seal of the root canal system.

For the new bioceramic sealers and cements, long-term follow-up studies are advocated.
